# Increase in imported malaria in the Netherlands in asylum seekers and VFR travellers

**DOI:** 10.1186/s12936-017-1711-5

**Published:** 2017-02-02

**Authors:** Brechje de Gier, Franciska S. T. Suryapranata, Mieke Croughs, Perry J. J. van Genderen, Monique Keuter, Leo G. Visser, Michele van Vugt, Gerard J. B. Sonder

**Affiliations:** 1 Department for Early Warning and Surveillance, Center for Epidemiology and Surveillance of Infectious Diseases, National Institute for Public Health and the Environment, Bilthoven, The Netherlands; 2 Department of Infectious Diseases, Public Health Service (GGD) of Amsterdam, Nieuwe Achtergracht 100, PO Box 2200, 1000 CE Amsterdam, The Netherlands; 3 National Coordination Centre for Travellers’ Health Advice (LCR), Nieuwe Achtergracht 100, PO Box 1008, 1000 BA Amsterdam, The Netherlands; 4 Department of Environment, Public Health Service (GGD) Hart voor Brabant, Ringbaan West 227, 5037 PC Tilburg, The Netherlands; 5 Department of Clinical Sciences, Institute of Tropical Medicine, Antwerp, Belgium; 6 Institute for Tropical Diseases, Harbour Hospital Rotterdam, Haringvliet 72, 3011 TG Rotterdam, The Netherlands; 7 Nijmegen Institute for International Health, Radboud University Nijmegen Medical Centre, Nijmegen, The Netherlands; 8 Department of Infectious Diseases, Leiden University Medical Centre, C5P46, Albinusdreef 2, 2333 ZA Leiden, The Netherlands; 9 Department of Internal Medicine, Division of Infectious Diseases, Tropical Medicine and AIDS, Academic Medical Centre, Meibergdreef 9, 1105 AZ Amsterdam, The Netherlands

**Keywords:** Epidemiology, Surveillance, Travel

## Abstract

**Background:**

Malaria is a notifiable disease in the Netherlands, a non-endemic country. Imported malaria infections occur regularly among travellers, migrants and visitors. Surveillance data were analysed from 2008 to 2015. Trends in amounts of notifications among risk groups were analysed using Poisson regression. For asylum seekers, yearly incidence was calculated per region of origin, using national asylum request statistics as denominator data. For tourists, denominator data were used from travel statistics to estimate incidence per travel region up to 2012.

**Results:**

A modest increase in overall imported malaria notifications occurred in 2008–2015 (from 222 in 2008 to 344 in 2015). Notably, in 2014 and 2015 sharp increases were seen in malaria among travellers visiting friends and relatives (VFR), and in asylum seekers. Of all *Plasmodium falciparum* infections, most (1254/1337; 93.8%) were imported from Africa; 1037/1337 (77.6%) were imported from Central and West Africa. Malaria in VFR was mostly caused by *P. falciparum* infection after visiting Ghana (22%) or Nigeria (19%). Malaria in asylum seekers was mostly caused by *Plasmodium vivax* infection from the Horn of Africa. The large number of notifications in asylum seekers resulted from both an increase in number of asylum seekers and a striking increase of malaria incidence in this group. Incidence of malaria in asylum seekers from the Horn of Africa ranged between 0.02 and 0.3% in 2008–2013, but rose to 1.6% in 2014 and 1.3% in 2015. In 2008–2012, incidence in tourists visiting Central and West Africa dropped markedly.

**Conclusions:**

Imported malaria is on the rise again in the Netherlands, most notably since 2013. This is mostly due to immigration of asylum seekers from the Horn of Africa. The predominance of *P. vivax* infection among asylum seekers warrants vigilance in health workers when a migrant presents with fever, as relapses of this type of malaria can occur long after arrival in the Netherlands.

**Electronic supplementary material:**

The online version of this article (doi:10.1186/s12936-017-1711-5) contains supplementary material, which is available to authorized users.

## Background

Malaria is caused by *Plasmodium* parasites and is transmitted to humans through the bites of infected female mosquitoes. Most malaria cases and deaths occur in sub-Saharan Africa. Other regions at risk are in Asia, Latin America and the Middle East. In 2015, 95 countries and territories had ongoing malaria transmission [1]. The World Health Organization (WHO) has declared the Netherlands malaria free since 1970. Nowadays malaria in the Netherlands occurs only as an imported disease in returning travellers or in visitors from endemic countries. Malaria is a mandatory notifiable disease in the Netherlands. Surveillance of imported malaria is used to monitor trends and as a feedback tool on the effectiveness of pretravel health consultation and indications for malaria chemoprophylaxis. The national coordination centre for traveller’s health (LCR) is responsible for the national travel medicine guidelines for the Netherlands. An earlier study found a decreasing trend of incidence of imported malaria in the Netherlands during the time-period 2000–2007 [2]. The annual number of imported malaria infections fell from 535 in 2000 to 197 in 2007. Most recorded infections were caused by *Plasmodium falciparum* (2131/2847; 75%), and most cases had recorded Africa as the most likely continent of infection (2068/2847; 82%). Almost half of all 2847 cases were travellers visiting friends and relatives (VFR) in Middle and West Africa. During this time period, malaria notifications among VFR had decreased as well (from 210 in 2000 to 77 in 2007) [2]. A decline in malaria incidence is seen globally; the WHO estimates a fall of 37% among populations at risk between 2000 and 2015 [1]. The risk of imported malaria, however, depends on several factors: malaria endemicity at the destination, the season of travel to the destination, the number of travellers to risk areas, the behavior of the traveller and adherence to personal protective measures like insect repellents, adequate use of chemoprophylaxis, and efforts for vector control around the accommodation. The aim of this study is to analyse trends in malaria notifications in the Netherlands, to assess whether the decline has persisted since 2007 and to identify risk groups. In recent years, the number of Dutch travellers to malaria endemic countries has increased [3] and since 2014 large numbers of asylum seekers from the Horn of Africa have arrived in the Netherlands. Although the incidence among asylum seekers was not studied separately in the previous study [2], in light of the recent increase in refugees seeking asylum in the Netherlands, the additional aim is to quantify the incidence of imported malaria in asylum seekers.

## Methods

### Data sources

All malaria notifications received between 1 January 2008 and 29 March 2016 were extracted from the Dutch electronic national surveillance system (‘Osiris’). A ‘date for statistics’ was defined as the day of onset of symptoms, or if this date was missing; the day of laboratory confirmation of malaria infection, or if this date was also missing; the day of notification. Because of possible delays in notification, all notifications received between 1 January 2008 and 29 March 2016 with a date for statistics in 2008–2015 were included in the analysis.

### Definitions

Whether a traveller was a VFR has been part of the notification questionnaire since July 2014. For notifications preceding this date or with missing data on reason for travel, a case was defined as VFR if the most likely country of infection matched the patient’s country of birth or the country of birth of either of the patient’s parents. Patients who were recorded as being asylum seekers or having visited the endemic country on business were excluded from the VFR definition, as were residents from endemic countries visiting the Netherlands.

For grouping countries of malaria infection, subcontinents were categorized according to the composition of macro-geographical regions described by the United Nations Statistics Division [4], with the following modifications: Zambia was included in Central and West Africa, whereas China, Yemen and Madagascar were included in South and Central Asia (termed South Asia in reference 2). Papua New Guinea was included in South East Asia. The United Nations Statistics Division describes the following regions: Africa (Eastern, Middle, Northern, Southern, Western), Americas (Northern, Caribbean, Central, South), Asia (Central, Eastern, Southern, South-Eastern, Western), Europe (Eastern, Northern, Southern, Western), Oceania (Australia and New Zealand, Melanesia, Micronesia, Polynesia). For grouping countries of origin of asylum seekers, an additional category was defined for the Horn of Africa, here defined as Eritrea, Ethiopia, Somalia, and Sudan. Sudan was included in the Horn of Africa category because this was often reported as the most likely country of infection for asylum seekers from Eritrea or Ethiopia.

### Absolute number of imported malaria, 2008–2015

To evaluate trends, absolute numbers were used in specific risk groups (VFR or business or study travellers). During the period of 2008–2015, it was not possible to calculate incidence for these groups, because reliable data on travel statistics of the non-Dutch speaking population, and business or study travellers were not available.

### Incidence of imported malaria in tourists, 2008–2012

During the period of 2008–2012, incidence in tourists was calculated by using denominator data per country of travel from the Continuous Holiday Survey (NIPO), which were at the time of this study only available up to 2012 [3]. This survey collects travel data from a random sample of the Dutch population four times a year. Participants complete a comprehensive questionnaire related to travel and holiday destinations over the telephone. The results are weighted to represent the general Dutch population. The questionnaire from the Continuous Holiday Survey is in Dutch, so only Dutch speaking citizens can participate. Because it is known that a large group of mainly first generation immigrants in the Netherlands do not speak Dutch, these data were only used to calculate incidence in tourists to minimize bias.

### Incidence of imported malaria in asylum seekers, 2008–2015

During the period of 2008–2015, incidence of imported malaria in asylum seekers were calculated. Data on total number of asylum seekers arriving in the Netherlands between 2008 and 2015 was kindly provided by the Central Agency for the Reception of Asylum Seekers (COA). The country of origin of asylum seekers registered by COA was used in the analysis to determine denominators of asylum seekers per world region.

### Analysis

Poisson regression was used to test trends in notifications. Coefficients were reported as incidence rate ratios, but for analyses which pertain numbers of notifications per year (without denominators), please note that ‘incidence’ refers to the total number of national notifications (per risk group). In Poisson regression analyses where denominator data was available, this was added to the model as ‘exposure’. Analyses were performed in STATA 13.0.

## Results

### Characteristics and absolute number of imported malaria, 2008–2015

**Fig. 1:**
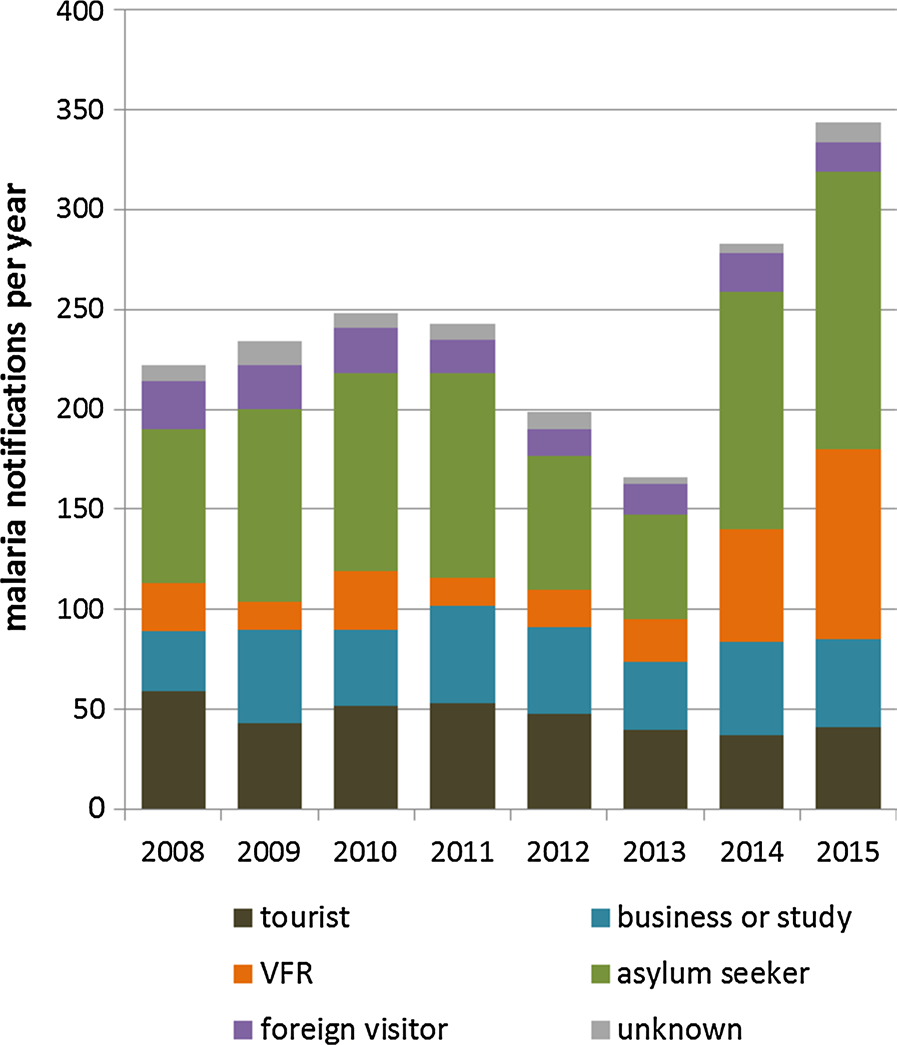
Malaria notifications 2008–2015 by reason for travel

A total of 1941 malaria notifications with a date for statistics during the time-period 2008–2015 were received by the National Institute for Public Health and the Environment. In 307/1941 (15.8%) cases the day of laboratory confirmation was used as date for statistics, as day of onset of symptoms was missing. In a further 12/1941 (0.6%) cases, the day of laboratory confirmation was missing as well, and the day of notification was used for statistics. The median timespan that elapsed from onset of symptoms to notification was 10 days (range 0–324). Figure 1 shows the absolute number of imported malaria per year and also shows malaria notifications stratified by reason for travel (Fig. 1). Analysis for secular trend over the whole study period up to 2015 showed a small but significant increase in malaria notifications (IRR 1.04 per year (95% CI 1.02–1.06, p < 0.001) for all species combined). However, time series analysis showed very slight downward trends up to 2013 in all malaria notifications combined (IRR per year 0.95, 95% CI 0.92–0.98, p 0.002). Among tourist travellers, a slight decline in absolute numbers of imported malaria was seen over the whole study period up to 2015 (IRR per year 0.95, 95% CI 0.91–0.99). Malaria cases among VFR increased markedly since 2013 (IRR 2.01 per year, 95% CI 1.63–2.46, p < 0.001). Among business or student travellers, yearly malaria notifications were stable (IRR 1.02, 95% CI 0.98–1.07). Additional file 1 shows regions of infection of Dutch travellers stratified by travel purpose.

The majority of notified malaria cases (1337/1941; 68.9%) was caused by *P. falciparum* infection, of which most (1254/1337; 93.8%) were imported from Africa; 1037/1337 (77.6%) were imported from Central and West Africa. Most *P. falciparum* cases among VFR occurred after travel to Nigeria (19%) and Ghana (22%). There has been a significant increase in *Plasmodium vivax* notifications in recent years (from 16 in 2013 to 129 in 2015). The majority of the 372/1941 (19%) imported *P. vivax* infections in the time period 2008–2015 originated from the Horn of Africa (214/372; 57.5%), rather than Asia (107/372; 28.8%) or South America (12/372; 3.2%). A further 129/1941 (7%) cases of *Plasmodium ovale*, 47/1941 (2%) cases of *Plasmodium malariae* and one *Plasmodium knowlesi* infection were reported in 2008-2015. Mixed infections, most often infections by both *P. falciparum* and *P. vivax*, were reported in 13/1941 (0.7%) of cases. The causative species was unknown in 42/1941 (2%) malaria notifications. Table 1 shows the annual number of imported infection by *Plasmodium* species (Table 1).

**Table 1:** Notified infections of malaria in the Netherlands, 2008–2015

Plasmodium species	2008	2009	2010	2011	2012	2013	2014	2015	Total
*P. falciparum*	178	187	185	175	142	128	163	179	1.337
*P. vivax*	20	28	32	33	30	16	84	129	372
*P. ovale*	14	14	19	18	15	13	17	19	129
*P. malariae*	4	1	8	12	7	6	5	4	47
Species unknown	4	1	2	4	3	3	16	9	42
Mixed infection	2	2	2	1	2	0	0	4	13
*P. knowlesi*	0	1	0	0	0	0	0	0	1
Total	222	234	248	243	199	166	285	344	1.941

Hospitalization occurred in 53.3% of all notified malaria cases and 7 deaths were notified. As all notified deaths occurred after *P. falciparum* infection, the case fatality rate for *P. falciparum* was 0.5%. While in 76/1941 (3.9%) of notifications the country of infection was unknown, also 8/1941 (0.4%) malaria cases were acquired in non-endemic countries. Among them was one case of congenital vivax malaria in a baby born in the Netherlands to a mother who had fled from Eritrea, a case of quartan malaria acquired through blood transfusion and one clinical trial participant who had been lost to trial follow-up after experimental malaria infection in another European country. Five other cases of *P. falciparum* infection reported to be acquired in non-endemic countries may have been acquired in other countries than reported, may have been cases of ‘airport or luggage malaria’ or might have been relapses of misdiagnosed *P. vivax* or *P. ovale* infections [5]. A map showing the countries of origin of imported malaria infections is found in Additional file 2.

### (Self-reported) adherence to chemoprophylaxis in Dutch resident malaria patients

In 2008–2015, 28/1941 (1.4%) cases of *P. falciparum* malaria were notified in Dutch resident travellers despite self-reported use of chemoprophylaxis according to the Dutch guidelines. Delayed onset of symptoms for *P. vivax* or *P. ovale* occurred in 53/1941 (2.7%) patients after using chemoprophylaxis according to the guidelines, which may be expected: *Plasmodium* species with persistent liver stages can establish infection despite adequate use of chemoprophylaxis [6]. One out of the 7 deceased patients took chemoprophylaxis (atovaquone/proguanil), but not according to the guidelines. The other 6 deceased patients did not take any prophylaxis. One of these was a Sudanese immigrant, all others were born in the Netherlands and above the age of 50. Five out of the six deceased Dutch travellers died of *P. falciparum* malaria after visiting The Gambia, which has been described before as a particular risk country [7, 8].

Whenever a notification of *P. falciparum* malaria despite chemoprophylaxis is received, the LCR investigates whether there is concern for resistance to prophylactic drugs in malaria parasites. In the majority of these cases, patients report illness with diarrhea or vomiting during the prophylactic regimen, forgetting to take their drug adequately, or taking atovaquone/proguanil with a possibility of diminished bio-availability [9]. In cases where no plausible explanation for chemoprophylaxis failure was found, hospitals were asked for the thick smear for sequencing of the parasite. Unfortunately, none of these samples had been stored.

### Incidence of imported malaria in tourists, 2008–2012

**Fig. 2:**
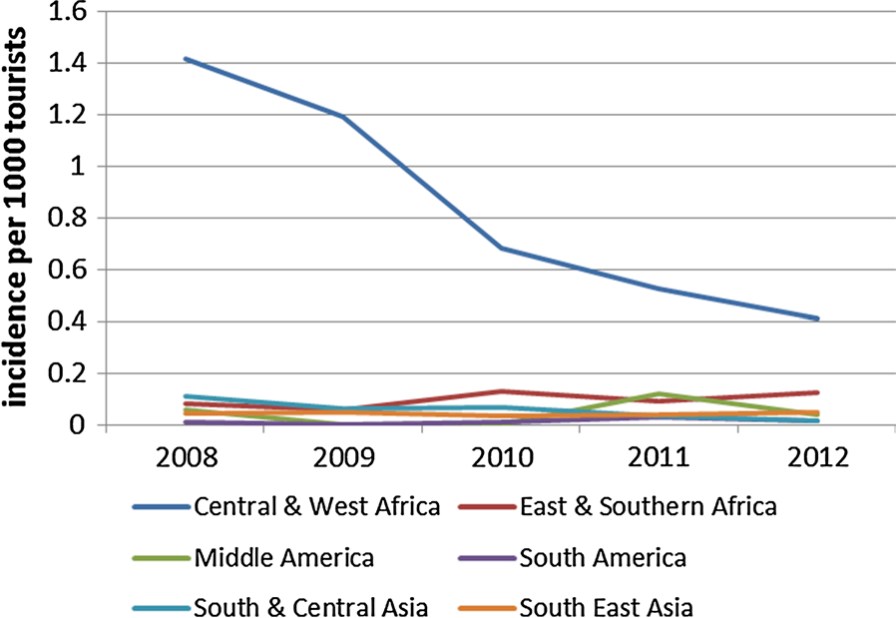
Incidence of malaria among tourists, by region of travel, 2008–2012

Figure 2 shows the incidence of malaria among tourist travellers in 2008–2012 (Fig. 2). A striking decrease in incidence of imported malaria originating from Central and West Africa was observed (IRR per year 0.72, 95% CI 0.64–0.81, p < 0.001). A large increase in travel to this region combined with a decrease in notifications underlies this decline. For 2013–2015 denominator data were not available, however in this period the yearly amount of notified cases after a visit to Central or West Africa has remained stable (20 cases in 2013 and 24 in both 2014 and 2015).

### Incidence of imported malaria in asylum seekers, 2008–2015

**Fig. 3:**
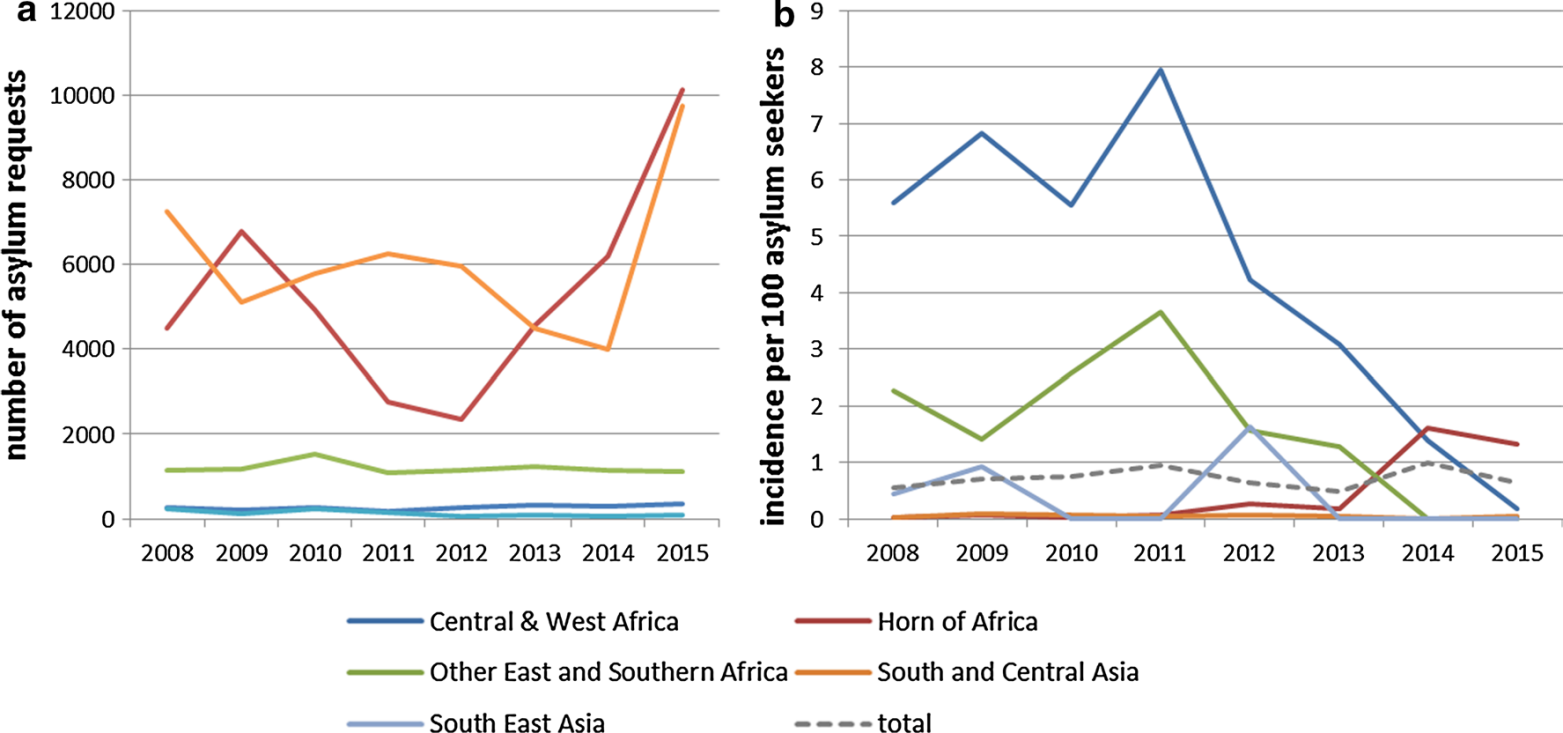
Total number of asylum requests per world region of origin (**a**) and malaria incidence among asylum seekers per world region of origin (**b**) 2008–2015

High numbers of malaria cases related to asylum seekers were notified in 2014 and 2015, this is shown in Fig. 1. Figure 3 shows the amount of asylum requests and malaria incidence among asylum seekers per world region of origin in 2008–2015. Of the 214/372 (57.5%) *P. vivax* cases originating from the Horn of Africa, 199/214 (93.0%) cases were among asylum seekers. Incidence of malaria in asylum seekers from the Horn of Africa ranged between 0.02 and 0.3% in 2008–2013 but rose to 1.6% in 2014 and 1.3% in 2015. Combined with the large number of asylum seekers from this region, this resulted in 234/259 (90.3%) of all malaria notifications in asylum seekers originating from the Horn of Africa in 2014–2015. Of these, 185/234 (79.1%) were specified as *P. vivax* infections and 66/234 (28.2%) cases were children <18 years. While asylum requests from Central and West Africa have remained stable, incidence of malaria in asylum seekers from Central and West Africa (which were mostly *P. falciparum* infections) has decreased drastically (IRR per year 0.80, 95% CI 0.77–0.84, p < 0.001). The overall incidence of malaria in asylum seekers from endemic regions remained stable between 0.5 and 1.0%.

## Discussion

### Absolute number of imported malaria, 2008–2015

This report describes and analyses trends in the notifications of imported malaria in the Netherlands during the period 2008–2015. The decrease described for the time-period 2000–2007 continued up to 2013, after which the decline in imported malaria infections seems to have stagnated. In the time period 2000–2007 the majority of the imported *P. vivax* infections originated from Asia (192/388; 55%) [2]. This is in contrast to the time-period 2008–2015, in which the majority of the imported *P. vivax* infections originated from the Horn of Africa (214/372; 57.5%). This was mainly due to the large numbers of *P. vivax* infections imported from the Horn of Africa in 2014 and 2015. In recent years, increases in imported malaria have mainly occurred among VFR from West Africa and in asylum seekers from the Horn of Africa. The recent increase in malaria notifications in VFR travellers might largely be attributable to an increase in visits to Central and West Africa (in particular Nigeria and Ghana). This is in contrast to the previous study of van Rijckevorsel et al. [2] that found a decrease in malaria notifications among VFR. In the same study information on the ethnic origin of the patients was available in 2141/2847 (75%) of the records. Of these patients almost half (1042/2141; 49%) were persons of Middle and West African origin, mainly from Ghana and Nigeria, of which 98 and 95% acquired the infection in the country of origin, respectively [2]. These findings are comparable to the results in this study.

### (Self-reported) adherence to chemoprophylaxis in Dutch resident malaria patients

Considering the number of malaria cases among Dutch travellers not using chemoprophylaxis (N = 845), improvements can still be made in reaching travellers for pre-travel health advice. This has shown to be especially difficult with last-minute holiday bookers and VFR [10]. The majority of malaria cases reported not having used chemoprophylaxis according to national guidelines. Also, there is no presumption of resistance of *P. falciparum* to chemoprophylaxis, although the evidence is weak. Therefore, alternative options for protecting travellers against malaria in intermediate risk areas such as stand-by treatment may improve uptake and adherence, both for tourists and VFR [11]. The main goal of prescribing chemoprophylaxis is to prevent the traveller to succumb after a *P. falciparum* malaria infection. Recent studies have shown both pre-travel consultation and compliant use of chemoprophylaxis to be associated with lower severity of malaria [12, 13]. Of the seven deceased malaria cases reported in the Netherlands since 2008, none used chemoprophylaxis according to the guidelines. However, the true number of malaria deaths among Dutch travellers is not known, as patients may have died abroad or after their case was notified.

### Incidence of imported malaria in tourists, 2008–2012

The most remarkable finding was the decreasing malaria incidence among tourists after travel to Central and West Africa (Figs. 1, 2). These observations may be the result of decreased prevalence of malaria in this region [14]. Adequate protective measures might also have contributed to the decrease seen in tourists.

### Incidence of imported malaria in asylum seekers, 2008–2015

The large number of asylum seekers from the Horn of Africa arriving in 2014–2015 in the Netherlands resulted in high rates of imported malaria. The analysis in this study show this is not solely due to an increased number of asylum seekers, but also to an increased number of imported malaria cases among this risk group as the incidence in this group also increased. These cases were mostly caused by *P. vivax* infection. A recent increase in *P. vivax* malaria in Eritrean asylum seekers has also been observed in other European countries [15, 16]. The estimated incidence in asylum seekers from the Horn of Africa is in line with the incidence in Eritrean asylum seekers in Sweden estimated by Sondén et al. (1.6% in 2014 in the Netherlands compared to 1.9% in the first months of 2014 in Sweden) [15]. The incidence of *P. vivax* in migrants from the Horn of Africa does not necessarily reflect the local epidemiology [17, 18], although a recent report from Ethiopia does support a predominance of *P. vivax* in this region [19]. Alternatively, migrants harbouring a *P. falciparum* infection may either succumb to the complications of the infection or be successfully treated on the migratory route, whereas symptoms of *P. vivax* may present later upon arrival in the Netherlands due to dormancy or relapse. In addition, it has been suggested that refugee camps might form zones of enhanced *P. vivax* transmission [15]. Incidence of malaria in asylum seekers from Central and West Africa has shown a drastic decline.

## Limitations

This study has several limitations. The results are dependent on the quality of the data provided in the notification questionnaire. It is uncertain to what extent risk groups such as asylum seekers or VFR are correctly classified. However, logical checks were used for this classification such as comparing country of birth to country of infection. Also, some misclassification may have occurred for the species of *Plasmodium*, as the microscopic determination of parasite species requires specific expertise and trained analysts. In addition, rapid antigen tests not distinguishing all species are increasingly used. Extensive clinical features were not included in the notification questionnaire. Clinical information other than hospitalization or death was not available.

The calculation of malaria incidence in asylum seekers by using the asylum requests per year also has its limitations: *P. vivax* malaria may present itself in a different year than the asylum request. Moreover, most people requesting asylum in a certain year will not have resided in the Netherlands for the entire year, therefore they may have had clinical malaria in months prior to entering the country.

The lack of denominator data for Dutch travellers divided by risk group (business, study and VFR travellers) precludes any conclusions about incidence of malaria in these groups. The increase in notifications of VFR cases to Central and West Africa may, therefore, be the result of an increase in total number of VFR visits, perhaps influenced by the subsidence of the economic crisis. Possibly, the reduced local prevalence influenced risk perception and preventive behaviors among VFRs. However this hypothesis is not supported by a recent study from the United Kingdom showing that VFR travellers to Nigeria and Ghana have similar knowledge and risk perception as non-VFR travellers [20]. Alternatively, the addition of a specific VFR-question in the notification form since 2014 may have increased the number of reported travellers defined as VFR in the analysis. It is possible that for the period before the introduction of a VFR-specific risk category in the questionnaire, part of the VFR travellers were misclassified as asylum seekers (for cases where the risk group ‘immigrant’ was chosen). Such a misclassification would partly mitigate the decrease in incidence in asylum seekers from Central and West Africa, as well as the increase in notifications among VFR. Furthermore, many countries have only regional malaria endemicity, or strong seasonality of transmission [21]. Therefore, it is often uncertain to what extent travellers are truly at risk of infection (and should be in the denominator to determine incidence).

## Conclusions

In conclusion, this study shows the decreasing trend in imported malaria in the Netherlands since 2000 has not continued. While the incidence among tourists has continued to decrease up to 2012, the amount of notified malaria cases in Dutch travellers (mainly VFR) has increased since 2013. Simultaneously, an increase in number of asylum seekers from the Horn of Africa as well as an increase in malaria incidence in this population resulted in high numbers of malaria cases among asylum seekers in the Netherlands since 2013. The predominance of *P. vivax* infection among asylum seekers warrants vigilance in health workers when a migrant presents with fever, as relapses of this type of malaria can occur long after arrival in the Netherlands. The recent increase of malaria in VFR travellers shows that travel health clinics might still be inadequately reaching this specific group of travellers.

## Additional files

**Additional file 1.** Total number of imported malaria infections in Dutch resident travellers, 2008–2015, by year, reason for travel and subcontinent of infection.

**Additional file 2.** World map showing the total number of notified imported malaria cases in the Netherlands by (most likely) country of infection, 2008–2015).
