# Supplementary figures and images for: Increase in imported malaria in the Netherlands in asylum seekers and VFR travellers
Source: Malar J. 2017 Feb 2;16:60. doi: 10.1186/s12936-017-1711-5 (PMC5288937; doi:10.1186/s12936-017-1711-5)

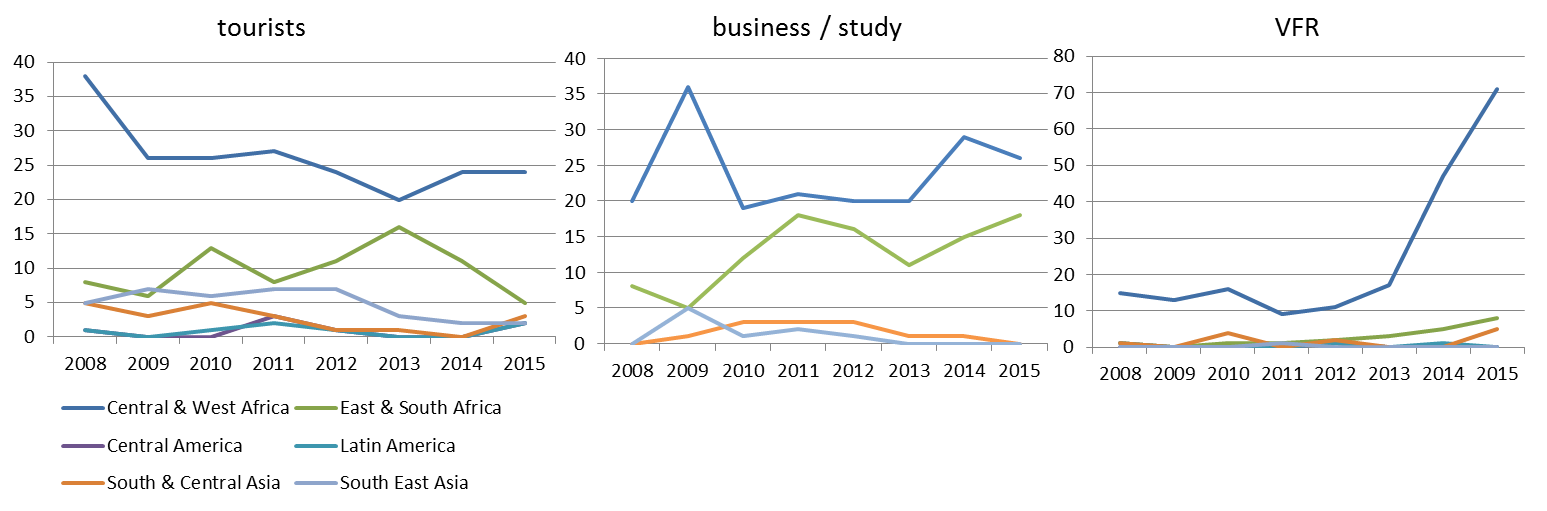

Supplement: Supplementary file 1 — Additional file 1. Total number of imported malaria infections in Dutch resident travellers, 2008–2015, by year, reason for travel and subcontinent of infection. [file 12936_2017_1711_MOESM1_ESM.png]

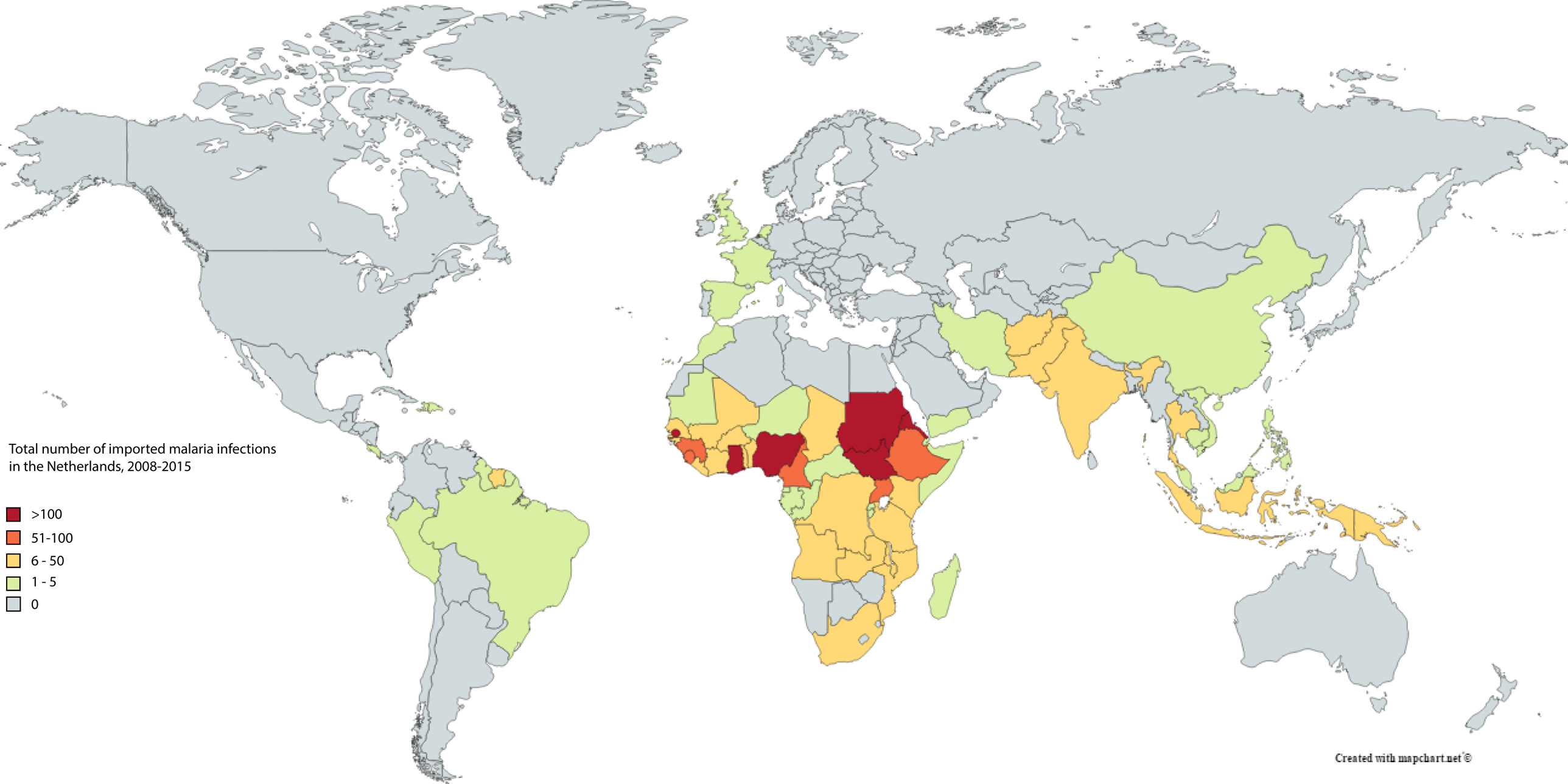

Supplement: Supplementary file 2 — Additional file 2. World map showing the total number of notified imported malaria cases in the Netherlands by (most likely) country of infection, 2008–2015). [file 12936_2017_1711_MOESM2_ESM.png]
